# 2894. PEP-in-Pocket (PIP): Long-Term Follow-Up of On Demand HIV Post-Exposure Prophylaxis

**DOI:** 10.1093/ofid/ofad500.165

**Published:** 2023-11-27

**Authors:** Maxime J Billick, Karla Fisher, Samantha Myers, Darrell Tan, Isaac Bogoch

**Affiliations:** University of Toronto, Toronto, Ontario, Canada; Toronto General Hospital, Toronto, Ontario, Canada; Unity Health Toronto, Toronto, Ontario, Canada; Division of Infectious Diseases, Department of Medicine, St. Michael's Hospital, Toronto, Ontario, Canada; Toronto General Hospital; University of Toronto, Toronto, ON, Canada

## Abstract

**Background:**

Pre-exposure prophylaxis (PrEP) and post-exposure prophylaxis (PEP) are well established methods of HIV prevention through the use of antiretroviral medications. However, the suitability of these tools for individuals with infrequent, higher-risk HIV exposures might be limited due to cost, high pill burden and/or barriers to care. PEP-in-pocket (PIP) involves prospectively identifying such individuals, proactively prescribing them 28 days of PEP medication, and providing instructions on when to self-initiate medications and how to follow up with care. We present long-term follow-up of a cohort of patients provided with PIP for HIV prevention.

**Methods:**

We evaluated the clinical characteristics and outcomes of patients using PIP for HIV prevention. Patients referred for PrEP or PEP care were offered PIP if they reported a low frequency (0-4 per year) of higher-risk HIV exposures of any type. The HIV prevention method was chosen through shared decision-making between patients and clinicians and was outside the realm of this study. Patients were followed at regular 4-6 months intervals.

**Results:**

We followed 112 patients prescribed PIP between the ages of 20-69 for a total of 183.8 patient-years. 108 (96%) patients were assigned male at birth. Thirty-five (31%) patients self-initiated a total of 69 courses of PIP during the observation period. Patients fluidly transitioned between HIV prevention modalities as circumstances warranted: 34 (31%) changed from PIP to PrEP, and 33 (30%) changed from PrEP to PIP. There were 18 episodes of bacterial sexually transmitted infections in 13 individuals (12%) using PIP. No HIV seroconversions were detected.

**Conclusion:**

PIP is an innovative HIV prevention strategy for individuals with a lower frequency of higher-risk HIV exposures, and provides patients with autonomy and agency over their care. Patients may transition between PIP and PrEP based on evolving risk. PIP should be included with PEP and PrEP as a biomedical HIV prevention option for individuals at risk for infection.

**Disclosures:**

**Darrell Tan, MD PhD**, Abbvie: Grant/Research Support|Gilead: Grant/Research Support|Glaxo Smith Kline: Grant/Research Support **Isaac Bogoch, MD, MSc**, BlueDot: Advisor/Consultant|NHL Players' Association: Advisor/Consultant

